# "Like Owner, Like Dog": Correlation between the Owner's Attachment Profile and the Owner-Dog Bond

**DOI:** 10.1371/journal.pone.0078455

**Published:** 2013-10-30

**Authors:** Marcello Siniscalchi, Carlo Stipo, Angelo Quaranta

**Affiliations:** Department of Veterinary Medicine, University of Bari "Aldo Moro," Bari, Italy; University of Rennes 1, France

## Abstract

During recent years, several studies have revealed that human-dog relationships are based on a well-established and complex bond. There is now evidence suggesting that the dog–human affectional bond can be characterized as an “attachment”. The present study investigated possible association between the owners' attachment profile assessed throughout a new semi-projective test (the 9 Attachment Profile) and the owner-dog attachment bond evaluated using a modified version of those used in studying human infants: Ainsworth's “strange situation”. The findings represented the first evidence for the presence of a correlation between the owners' attachment profile and the owner-dog attachment bond throughout procedure and behavioural analyses involving controlled observations.

## Introduction

Attachment and affectional bonds are close relationships that bind individuals together in time and space: they are emotionally relevant and characterized by providing care and protection and/or using the other as a source of security and comfort [1]–[6].

During recent years, several studies have revealed that human-dog relationships are based on a well-established and complex bond [7]–[10]. There is now evidence suggesting that the dog–human affectional bond can be characterized as an attachment: dogs show towards their owner attachment behaviours, which closely resemble those reported in human infants and chimpanzees [9]–[11]. The procedure and behavioural analyses used to investigate the dog-human attachment is a modified version of those used in studying human infants: Ainsworth's “strange situation”. The Strange Situation procedure involves conducting controlled observations of a subject's response to being placed in an unfamiliar room, introduced to an unfamiliar adult (the stranger) and subjected to three short episodes of separation/reunion from the attachment figure [8], [10], [12]. Previous studies, using the Strange Situation Procedure, showed that dogs' both behavioural and physiological response to stress (e.g. separation from their owner) is determined by experience and training together [8]. In addition the Strange Situation has been used to investigate differences between women and men owners during interactions with their dogs revealing differences in the use of verbal communication [12]. At present, however, most of the studies on human-dog relationship including the assessment of people's attachment towards their dogs are based on questionnaires and interviews [12], [13] that have previously been criticized since the owner's subjective assessment criteria increased the variability of results [14], [15].

The 9 Attachment Profile (9AP) is a new semi-projective test for assessing the quality of the interpersonal relationships based on self/other perception and internal working models of adult attachment [16], [17]. The use of a semi-projective test has broad advantages since the psychological variables scored are more difficult to be detected by human beings and consequently the possibility to manipulate the response is very little.

In the light of current evidence, the present study investigated possible association between the owners' attachment profile assessed throughout a new semi-projective technique (9AP) and the owner-dog attachment bond evaluated using the Strange Situation Test.

## Materials and Methods

### Ethics Statement

The experiments were conducted according to the protocols approved by the Italian Minister for Scientific Research in accordance with EC regulations; in addition, before the experiment began, the procedure was explained to owners and written informed consent was obtained.

### Subjects

Twenty-nine owner-dog pairs participated in this study. Of these, 4 dyads had to be excluded from the sample due to technical problems (e.g. deviation from the instructions by the dog's owner). The final sample contained 25 owner-dog pairs. The owners were 13 women and 12 men ranged from 16 to 48 years (28.33±2.01; mean years ± s.e.m.). The dogs were 11 females and 14 males of various breeds (2 Dachshund, 1 Maltese, 1 Cavalier King Charles Spaniel, 2 Boxer, 1 German Shepherd and 18 mongrel dogs). Dogs ranged from 1 to 11 years of age (3.86±0.72 mean years±s.e.m.). All dogs were pets living in households and spent their entire lives with the same owner. Seven females and four males were neutered. No subject had been tested previously. All the participants were volunteers recruited by means of public advertising in veterinary hospitals and faculty of veterinary science notice boards. The same man, who had never met owner-dog pairs before the day of the test, played the role of the stranger.

### Experimental setup

**Figure 1:**
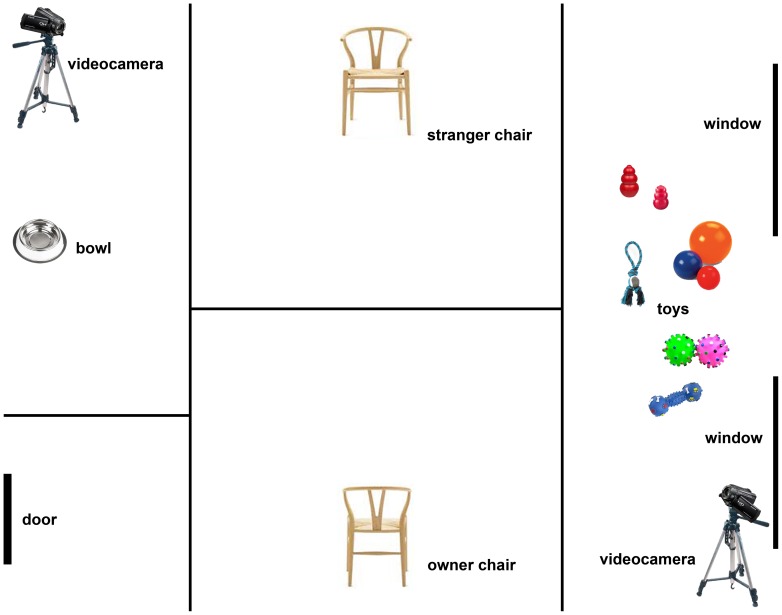
Experimental setup. Schematic representation of the testing apparatus.

The experiment was carried out at the Faculty of Veterinary Medicine of Bari University, Italy in a bare room (3.5 m × 4.5 m) isolated from the rest of the building to avoid any noise interference. The testing area was the same for all subjects and was similar as possible to the controlled environment described in the Ainsworth's strange situation (see Fig.1). The room was equipped with two chairs, different dog toys (three balls of different sizes, two kong-food toy of small and medium sizes, a rope pull-toy, and three squeaky toys) and a water bowl. Dog-owner dyads behaviour during the experiment was video-recorded using two digital video cameras (Sony handicam HDR-XR550) placed at two adjacent corners (see Fig. 1) of the room to extend the video recording area. One of the two video cameras was connected with a monitor positioned outside the room to observe independently the experimental sessions.

### Procedure

The experimental procedure used was similar to that used in previous studies [10]. The detailed description of the "Strange situation" procedure is reported in Table 1.

**Table 1:** Description of the Strange Situation procedure (modified by E. Prato-Previde, G. Fallani and P. Valsecchi, 2006).

Episode 1: owner and dog	The owner sat quietly and the dog was free to explore the room.
Episode 2: owner, dog and stranger	The stranger entered the room, sat quietly for 1 min, conversed with the owner for the second minute, approached the dog and attempted to stimulate play during the last minute. At the end of this episode the owner left the room unobtrusively.
Episode 3: stranger and dog (1^st^ separation episode)	The stranger continued to play with the dog if it was willing; if it was inactive or distressed, the stranger attempted to distract it with play or by providing verbal and tactile comfort.
Episode 4: owner and dog (1^st^ reunion episode)	The owner entered the room and greeted and/or comforted the dog as usual after returning from work or shopping. The stranger quietly exited the room. The owner had been told that she/he was free to play with the dog throughout the episode. At the end of this episode the owner left the room.
Episode 5: dog alone (2^nd^ separation episode)	The dog remained alone for 3 min, but was constantly observed by the owner and researchers on the monitor in the adjacent room.
Episode 6: stranger and dog	The stranger entered the room and followed the same protocol as in episode 3.
Episode 7: owner and dog (2^nd^ reunion episode)	The owner entered the room greeted the dog as though he/she had just returned from work or shopping. The stranger left the room unobtrusively.
Episode 8: dog alone (3^rd^ separation episode)	The dog remained alone and was monitored as in episode 5.

Briefly, the day of the experiment each dog-owner dyad was conducted first to a room adjacent to the experimental one and the procedure was described to the owner (introductory episode); in addition the owner was asked to behave towards their dog as spontaneous as possible during the whole experiment to avoid any possible modification of dog's behaviour due to both the unfamiliar environment and the strange situation.

The introductory episode was followed by 8 three-minute episodes in which the dog was placed inside the experimental room, introduced to the stranger and subjected to three episodes of separation/reunion with their owner.

### Attachment profile questionnaire – 9AP

Fifty-one owners were asked to fill in a questionnaire aimed to evaluating their attachment profile (9 Attachment Profile). The 9 Attachment Profile (9AP) [16] is a semi-projective test for assessing the quality of the interpersonal relationships based on self/other perception and internal working models of adult attachment. This test consists of seven basic pictures; each picture represents a situation with one black figure and one or more white figures in different environments. Two equal lists of nine differential semantics anchored by opposed terms accompany the presentation of each picture. In the first list, participants rate on 9-point scale for each differential semantic their self-perception; in the second list their perception of the others. 9AP provides eighteen bipolar scales regarding psychological and emotional constructs, nine self-related and nine other-related: Acceptance—Rejection, Friendliness—Hostility, Power—Submission, Security—Insecurity, Availability—Unavailability, Calm—Agitation, Satisfaction—Dissatisfaction, Independence—Dependence, Lack of competition—Competition. Higher scores correspond to the first term of each bipolar scale, lower scores to the second term.

The results of the 9AP were subsequently analyzed by means of software developed by Dr. Filippo Desantis commonly used in clinical psychology to asses humans attachment profiles and different types of owner's attachment profiles were subsequently divided into four categories: confident, disorganized (not-confident), avoidant (not-confident), anxious (not-confident).

### Behavioural score

Two trained observers using a continuous recording method analyzed the video recorded behaviour of each dog during the experimental episodes. Inter observer reliability was assessed by means of independent parallel coding of a random sample of video recorded sessions (i.e., 45%) and calculated as percentage agreement; percentage agreement was always more than 94%. According to the procedure followed in previous studies [10] a total of 18 behaviours were recorded. The occurrence of each behaviour was calculated as a proportion of the total number of events continuously scored during episodes. To achieve normality, the proportions were arcsine transformed using Bartlett's correction for continuity.

GLM analysis for repeated measures were carried out with groups (two levels resulting from 9AP questionnaire: confident-owner, not-confident-owner) and owner gender as between-subjects factor, and time (seven levels: episode 1 to episode 7) as within-subjects factor. To break down interactions, contrasts were performed comparing the proportion of occurrence of each behavioural category during every single episode to average proportion of behavioural occurrences during all episodes across dogs bonded with confident (CO) and not-confident (N-CO) owners.

Furthermore, to detect differences in behaviour towards the owner and the stranger a second ANOVA for repeated measures was carried out comparing episodes characterized by the exclusive presence of the owner (episodes 4+7) with those characterized by the exclusive presence of the stranger (episodes 3+6) or by isolation (episodes 5+8). For all ANOVAs, Fisher's Protected LSD test was carried out to detect differences in single comparisons. For all statistical tests, SPSS software (SPSS Inc., Chicago, IL, U.S.A.) was used, and the results were considered statistically significant for P<0.05.

## Results

Since behavioural results related to owner gender obtained in this research matched those of the previous one [12] to avoid unnecessary repetition and overlapping of information, we decided to present here only those results most characterizing the groups of dogs related to owners' results of the 9AP.

### Attachment profile questionnaire – 9AP

On the basis of subjective differences in the results of the 9AP questionnaire, owners were divided into two categories: “confident” (n = 41) and “not-confident” (n = 10).

A subsample of 25 owner-dog dyads “confident” (n = 15: 8 Female owners: 25.46±3.32; mean years ± s.e.m.; 7 Male owners: 29.16±2.12; mean years ± s.e.m.), “not-confident” (n = 10: 5 Female owners: 29.36±2.52; mean years±s.e.m.; 5 Male owners: (26.28±3.04; mean years±s.e.m.) was subsequently tested in the Strange Situation Test.

The GLM analysis for repeated measures revealed the presence of several statistical significant differences in behaviour between dogs bonded with confident (CO) and not-confident (N-CO) owners.

### Exploration

**Figure 2:**
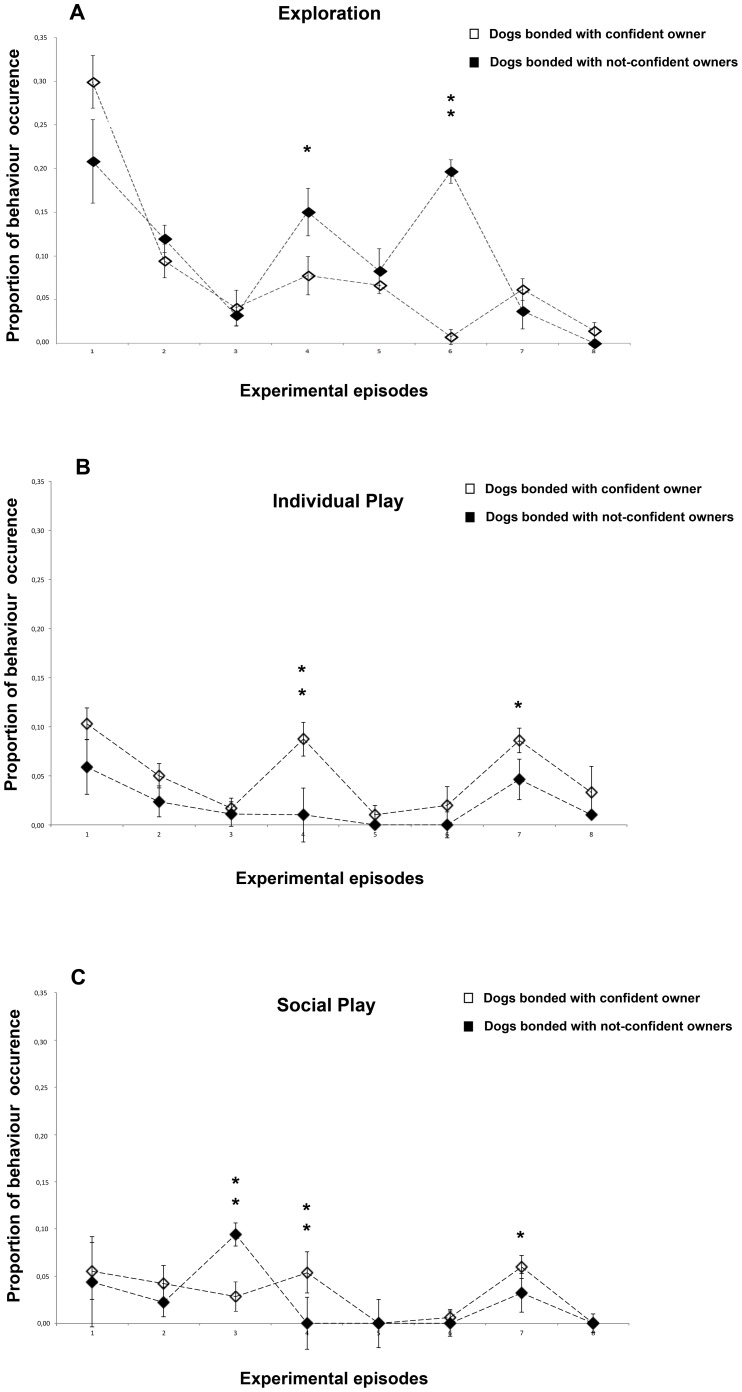
Behavioural categories that indicate secure base effect. a) Exploration, b) Individual play, c) Social play. Proportion of behavioural occurrences in the two groups of dogs across the 8 experimental episodes (group means with SEM are shown). Asterisks indicate significant differences between dogs' groups (see text for details) : *P<0.05; **P<0.01.

Different patterns of exploratory behaviour of the two groups of dogs during successive episodes are shown in Figure 2a.

According to the work of Prato-Previde et al. [10] exploration behaviour declined in all dogs sharply from episode 1 when the dog was alone with the owner to episode 2 when also the stranger was present remaining low in episode 3, when the dog was alone with the stranger.

Across the following episodes of the test, dogs differed significantly in exploration behaviour (F(7,161) = 18.212, P<0.001; Fig. 2a). In particular, in episode 4 when the owner entered the room, N-CO dogs engaged in exploration behaviours significantly more than CO dogs (episode 4: CO versus N-CO, P<0.01) and surprisingly this difference was greater in episode 6 when the stranger entered the room (episode 6: CO versus N-CO, P<0.001, see Fig. 2a). It is interesting to note, however, that at visual inspection of fig. 2a in CO dogs, as expected, the lowest proportions of sample points spent exploring were achieved during episodes characterized by owner absence (respectively episodes 3, 6 and 8) whereas this was not the case for (N-CO) dogs. A comparison between the overall proportion of sample points spent exploring in episodes characterized by owner (O) presence (1, 4, 7), stranger (S) presence (3, 6) or by isolation (I; 5, 8) revealed a significant difference across these situations (GLM ANOVA: F(2,46) = 14.637, P = 0.012 O vs. S vs. I, P<0.001). Overall, the dogs explored more in the presence of their owner than with the stranger or alone (post-hoc analysis Fisher's protected LSD: O vs. S, P<0.005, O vs. I, P<0.001); in addition the exploratory behaviour was higher during episodes characterized by stranger presence respect to isolation (S vs I, P<0.05). Finally, results revealed that (N-CO) dogs explored significantly more in the presence of the stranger than (CO) dogs (F(1,23) = 8.594, P = 0.008).

### Individual play

Results revealed that dogs played significantly different during episodes (F(7,168) = 7.535, P = 0.000) (Fig. 2b). Contrasts revealed that this difference was due to the fact that dogs played individually more during episode 1 (P<0.05) and less during episodes 3 (P<0.01), 5 (P<0.05) and 6 (P<0.01) respect to the average time spent playing individually during all episodes.

In particular, in episode 4 when the owner entered the room, (CO) dogs engaged in individual play significantly more than (N-CO) dogs (episode 4: CO versus N-CO dogs, P<0.01). A comparison between the overall proportion of sample points spent exploring in episodes characterized by owner (O) presence (1, 4, 7), stranger (S) presence (3, 6) or by isolation (I; 5, 8) revealed that a significant difference across these situations (GLM ANOVA: F(2,46) = 8.267, P<0.01). Overall, the dogs played individually more in the presence of their owner than with the stranger or alone (O vs S: P<0.01; O vs I: P<0.01).

### Social play

Figure 2c shows the proportion of sample points spent playing with both owner and stranger of CO and N-CO dogs: results revealed that N-CO dogs spent more time playing with the stranger during episode 3 (first separation episode from the owner) respect to CO dogs (F(1,23) = 16.896, P<0.001) whereas during both reunion episodes with the owner x social play behaviour was higher for CO dogs respect to N-CO dogs (episode 4: (F(1,23) = 9,389, P = 0.005); episode 7: (F(1,23) = 4,747, P = 0.040)

### Passive behaviour

GLM analysis for repeated measures revealed a significant main effect of episodes on passive behaviour (F(7,161) = 11.088, P<0.001). Contrasts revealed that dogs displayed passive behaviours less in the presence of the stranger than with their owner or alone (episodes 3 vs average passive behaviour: F(1,23) = 12.330, P = 0.002; episodes 6 vs. average time spent on passive behaviour during episodes: F(1,23) = 14.545, P<0001). Comparison between the overall proportion of sample points spent passive in the episodes characterized by owner presence [1], [4], [7], stranger presence [3], [6] or by isolation [5], [8] revealed that CO dogs are significantly less passive in the presence of the stranger respect to (N-CO) dogs (F(1,23) = 12.330, P = 0.002).

### Approach

The dogs approached the stranger differently during episodes (F7,161) = 7,153, P = 0.014): during separation episodes (N-CO) dogs approached the stranger more than (CO) dogs (episode 3: F(1,23) = 6,261, P = 0.020; episode 6: F(1,23) = 18,492, P = 0.000).

### Withdraw

As expected, avoidances responses occurred significantly more in the presence of the stranger during the two separation episodes (GLM: F(7,161) = 9.827, P<0.001; Post hoc test Fisher's Protected LSD: Episodes 3 and 6 vs. all other episodes P<0.001). No difference between CO and N-CO dogs were found.

### Oriented to person

**Figure 3:**
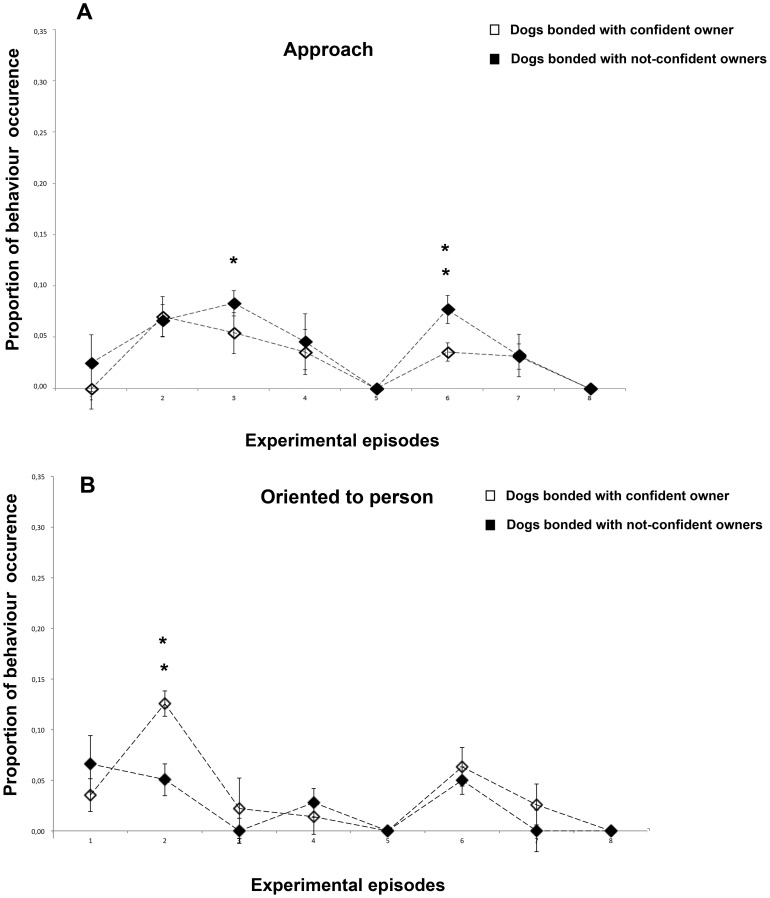
Behavioural categories that indicate proximity seeking. a) Approach, b) Oriented to person. Proportion of behavioural occurrences in the two groups of dogs across the 8 experimental episodes (group means with SEM are shown). Asterisks indicate significant differences between dogs' groups (see text for details) : *P<0.05; **P<0.01.

Dogs paid attention to the stranger significantly more respect to the average during episodes 2 F(1,23) = 26,901, P<0,001 and 6 F(1,23) = 7.095, P = 0.014. As shown in Fig. 3b, CO dogs paid more attention to the stranger than N-CO dogs when he entered the room for the 1st time in the presence of the owner (F(1,23) = 8.958, P = 0.006).

### Following

Most of the dogs (85%) followed their owner to the door at some point during the procedure (episodes 2, 4 and 7), (51%) of the sample followed the stranger to the door when he left the room during episodes 3 and 6 and finally only 4 dogs (16%) never showed following behaviour.

### Scratch door

**Figure 4:**
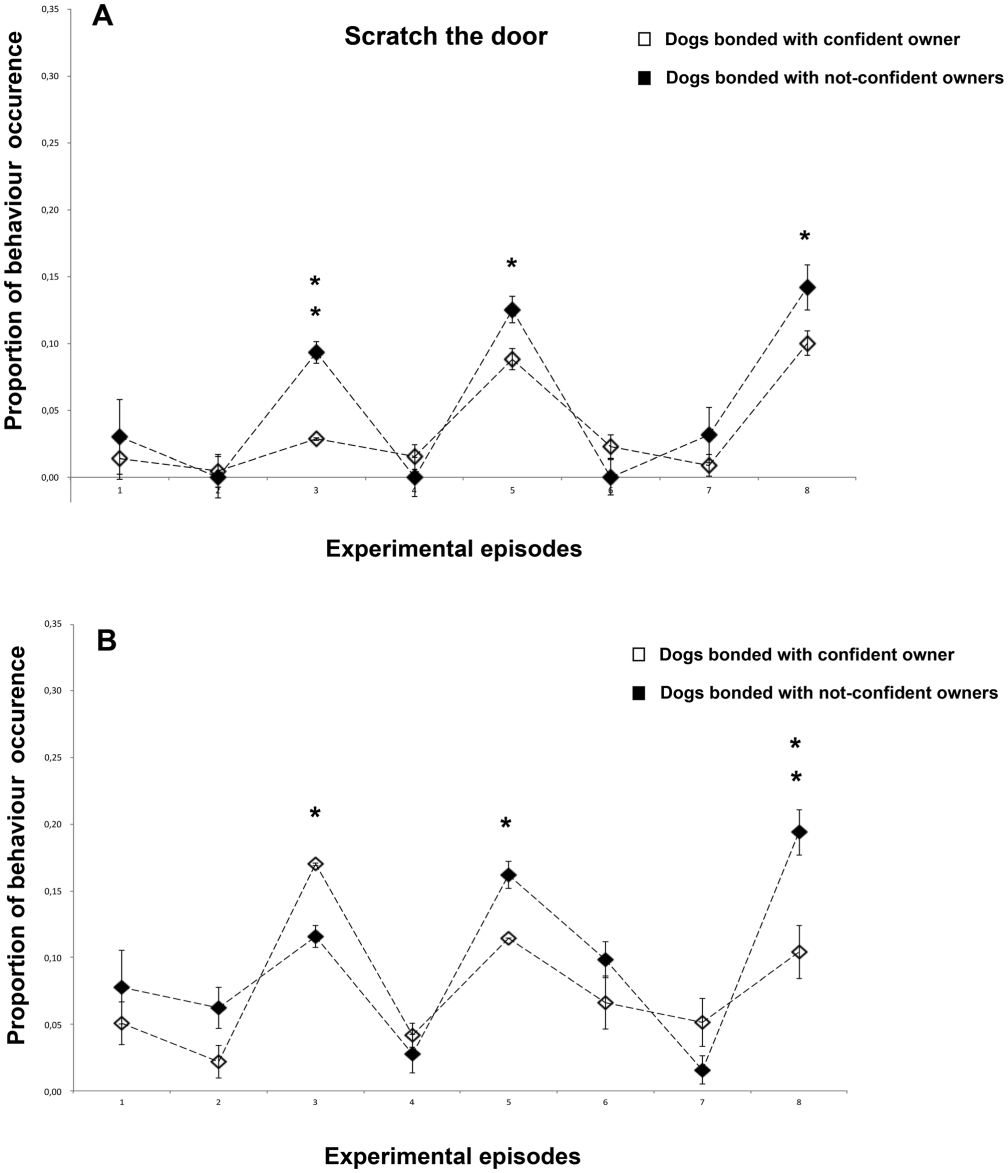
Behavioural categories that indicates searching. a) Scratch door, b) Oriented to door. Proportion of behavioural occurrences in the two groups of dogs across the 8 experimental episodes (group means with SEM are shown). Asterisks indicate significant differences between dogs' groups (see text for details) : *P<0.05; **P<0.01.

Dogs scratched the door significantly more during the 1st separation episode (ep. 3) (F(1,23) = 7.601, P = 0.011) and when they remained alone (ep. 5, 8) (ep. 5: F(1,23) = 44.296, P = 0.000; ep. 8: F(1,23) = 46.124, P = 0.000) (Fig. 4a). This behavioural pattern was enhanced for N-CO dogs respect to CO dogs (CO vs. N-CO dogs, episode 3: F(1,23) = 14.167, P = 0.001; ep. 5: F(1,23) = 6.158, P = 0.021; ep. 8: F(1,23) = 7.030, P = 0.014).

### Oriented to door

Oriented behaviour increased significantly during the absence of the owner (episodes 3,5,8) (F(7,161) = 4.223, P<0.001) (Fig. 4b). In addition, dogs bonded with confident owners stayed more oriented towards the door than dogs bonded with not-confident owners during the 1st separation episode (F(1,23) = 4.393, P<0.05), whereas the opposite behaviour was recorded when the dog was alone (episode 5:F(1,23) = 4.581, P<0.05), episode 8:F(1,23) = 18,217, P<0,001).

### Physical contact with person

**Figure 5:**
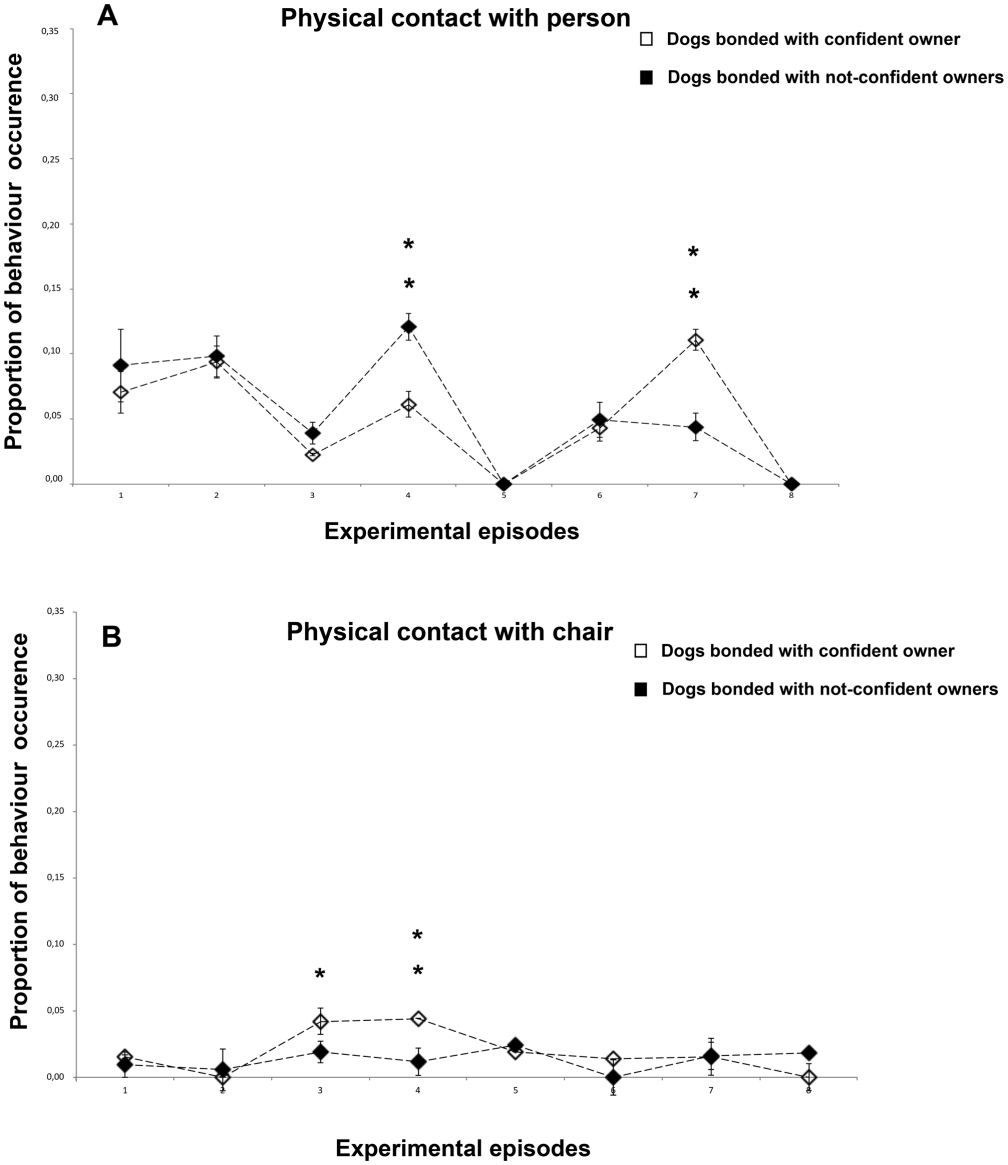
Behavioural categories that indicate comfort seeking. a) Physical contact with person, b) Physical contact with chair. Proportion of behavioural occurrences in the two groups of dogs across the 8 experimental episodes (group means with SEM are shown). Asterisks indicate significant differences between dogs' groups (see text for details) : *P<0.05; **P<0.01.

Subjects maintained physical contact with both the owner and the stranger mainly during episodes 2 (F(1,23) = 13,188, P = 0.001), 4 (F(1,23) = 7,708, P = 0.011) and 7 (F(1,23) = 11,496, P = 0.032) (Fig. 5a). Contrasts revealed that during the 1st reunion episode N-CO dogs maintained physical contact with their owner more than CO dogs (P<0.001) and unexpectedly this tendency reverted during the 2nd reunion episode (P<0,001). A comparison between the overall proportion of sample points spent exploring in episodes characterized by owner (O) presence (1, 4, 7) and stranger (S) presence (3, 6) revealed that dogs spent more time in physical contact with the owner (P<0.01).

### Physical contact with chair

The main results revealed that dogs were significantly more in contact with their owner's empty chair in the presence of the stranger (ep. 3: F(1,23) = 5.530, P = 0.028; ep. 6: F(1,27) =  7.833, P = 0.010) while with stranger empty chair during the 1st reunion episode (F(1,17) = 5.714, P = 0.029) (Fig. 5b). Furthermore, during the 1st separation episode CO dogs were significantly more in contact with their owner's empty chair respect to N-CO dogs (F(1,23) =  10,783, P = 0.004) while during the 1st reunion episode they were significantly more in contact with the stranger empty chair respect to N-CO dogs (F(1,23) =  10,157, P = 0.004).

### Vocalising

Vocal behaviour was low during the presence of the owner and increased significantly in the presence of the stranger and during social isolation (F(2,46) = 27,998, P = 0.000, O vs. S (P = 0.003); O vs. I (P = 0.00); I vs. S (P = 0.002). A comparison of vocal behaviour between CO and N-CO dogs revealed that N-CO dogs vocalized significantly more respect to CO dogs during the 1st encounter with the stranger in the presence of the owner (F(1,23) = 5.348, P = 0,034). *P = 0.002*

### Greeting

As expected, subjects showed significantly more levels of greeting behaviour towards the entering owner compared with the stranger (ep. 2,6 vs ep. 4,7: F(24) = 7.091, P<0.001). In addition CO dogs showed a significantly intense greeting behaviour towards their owner compared to N-CO dogs (ep. 4: F(1,23) = 12.337, P = 0.002; ep. 6: F(1,23) = 5.689, P = 0.026). Finally, N-CO dogs showed no greeting behaviour towards the stranger during the experiment.

## Discussion

Although It is widespread opinion that dog-owner relationship affected dog behaviour and recent research showed that dogs can learn socially from human beings [18]-[20], and engage in complex communication with people [21], [22] sharing very similar neurophysiological pattern to analyze emotion [23]–[25] at present there are no data examining whether the owner personality affects the behaviour of the dogs in a controlled situation. Here we reported for the first time the presence of a correlation between the attachment profile of the owners evaluated through a semi-projective test (the 9 Attachment Profile) and the owner-dog attachment bond evaluated using a modified version of that used in studying human infants: Ainsworth's “strange situation”. The use of a semi-projective test to evaluate owners' attachment profile has broad advantages since the psychological variables scored are more difficult to be detected by human beings and consequently the possibility to manipulate the response is very little [16]. The Strange Situation procedure, on the other hand, involves conducting controlled observations of dogs' response to being placed in an unfamiliar room, introduced to an unfamiliar adult (the stranger) and subjected to three short episodes of separation/reunion from the attachment figure. Ainsworth [5] stated that the secure base effect was a primary factor in identifying an attachment bond. Three measures of the secure base effect have been identified: 1) play and exploration become depressed when in the presence of just the stranger and when alone, but recover after reunion with the mother; 2) infant cease playing or exploring on the entrance of the stranger and return to their mother's side; and 3) infants will sometimes play with the stranger in their mother's presence, but not her absence.

In our experiment, the first main result was that when the owner entered the room after the 1^st^ separation episode (episode 4), dogs bonded with not-confident owners engaged in exploration behaviours significantly more than dogs bonded with confident owners and surprisingly this difference was greater when the stranger entered the room after the 2nd separation episode (episode 6). These results suggest that for (CO) dogs the owner provided a secure base for exploration and that the entrance of the stranger negatively affected exploratory behaviour, indicating stranger's wariness or fear; on the contrary, the behaviour of (N-CO) dogs suggested that they didn't have a strong structured secure base effect with their owner since the entrance of the stranger after the 2nd separation episode represented an event similar to the entrance of their owner (i.e. the contact with a human being represents a positive event after isolation independently from the attachment figure). This result was confirmed by the fact that during the 2nd separation episode (N-CO) dogs approached the stranger more than (CO) dogs. The second main result regarded play behaviour. In accordance with previous results (10), our sample of (CO) dogs engaged in social play significantly more in the presence of the owner respect to the stranger whereas (N-CO) dogs spent more time playing with the stranger during the first separation episode from the owner. This result suggested that for (CO) dogs the presence of the owner provided a sufficient sense of security to promote play with the stranger. Similarly, when the owner entered the room after the 1st separation episode, (CO) dogs engaged in individual play significantly more than (N-CO) dogs respect to the average time spent playing individually during all episodes. This confirmed that behaviour of (CO) dogs in the Strange Situation test resembled that of adult dogs in the work of Prato Previde et al. [10] and that of both human and chimpanzee infants [4], [11].

The third crucial point is that (CO) dogs also exhibited a range of behaviours, e.g. they stayed more oriented towards the door than (N-CO) dogs during the 1st separation episode (proximity seeking behaviour when separated from their owner), and showed a significantly intense greeting behaviour towards their owner compared to (N-CO) dogs which are behaviours well attested to establish the presence of an attachment. Interestingly a comparison of vocal behaviour between (CO) and (N-CO) dogs revealed that (N-CO) dogs vocalized significantly more respect to (CO) dogs during the 1st encounter with the stranger in the presence of the owner which underline that the presence of the owner for (N-CO) dogs not provided a sufficient sense of security to promote active interaction with the stranger (e.g. CO dogs paid more attention to the stranger than N-CO dogs when he entered the room for the 1st time in the presence of the owner); in this case the arousal state of dogs bonded with not confident owners was probably enhanced by the presence of the stranger and vocalizing behaviour might function to indicating distress.

In conclusion our findings reported an association between the owner's attachment profile assessed throughout a new semi-projective technique (9AP) and the attachment bond that dogs structured towards their owner evaluated using a modified version of those used in studying human infants attachment bond: the Ainsworth's Strange Situation Test.

In humans, the Attachment theory describes the genetic tendency of newborns to establish a close relationship (attachment) with individuals who are sensitive and responsive in social interactions with them (caregivers) especially during stressful situation (e.g. infantile needs for shelter, protection, security, food etc.) [26]; on the other hand, caregivers' behavioural responses are crucial to the development of different patterns of attachment and lead to internal working models “IWM” (i.e. the development and maintenance of mental representations of the self and others) which will guide the individual's perceptions, emotions, thoughts and expectations in later intra and inter-specific relationships [27]. As a consequence, a valid logical explanation for the association reported here between the owner's attachment profile and the owner-dog attachment bond is that the type of the attachment that the owner has structured during his early life with his caregiver (presumably the owner's parents) affects the owner-dog relationship throughout an unaware projection of the owner's internal working models on dog's behaviour.

Indeed, a particularly interesting aspect that deserves further investigation is the effect of early socialization with humans on the Strange Situation procedure since evidence exists of a decrease in the variability of the behavioural scores produced from scientific procedures for dogs that have had adequate socialization during their early lives [28]. Although none of the owners in our study reported that their dogs suffered any serious deficiencies in their social behaviour, we cannot totally exclude the possibility that a different degree of socialization could represent an alternative explanation to difference observed between CO and N-CO dogs (e.g. it could be possible that N-CO dogs were socialized differently and feel more comfortable with unfamiliar people - see more physical contact and play with stranger and higher exploration in his presence in N-CO dogs than in CO dogs).

Taken together these findings, although preliminary, are of interest because they represented the first evidence for a correlation between the owner's personality and the dog's behaviour under controlled experimental observations. Further research is clearly needed to explore other factors such as genetic, social or hormonal influences using a larger sample of owner-dog dyads.
